# Supplementary material for: Predicting the evolution of Escherichia coli by a data-driven approach
Source: Nat Commun. 2018 Sep 3;9:3562. doi: 10.1038/s41467-018-05807-z (PMC6120903; doi:10.1038/s41467-018-05807-z)
Supplement: Supplementary file 1 — Supplementary Information [file 41467_2018_5807_MOESM1_ESM.pdf]

Predicting the evolution of *Escherichia coli* by a data-driven approach

Wang et al.

SUPPLEMENTARY INFORMATION

Supplementary methods

Whole-genome re-sequencing and mutation discovery of evolved strains

Illumina HiSeq instrument generated 135,390,905 paired-end 100bp reads for 41 samples in a single lane. Among them, 110,003,665 reads of 35 samples pertained to this study. The average of total reads per sample was 3,142,961. The adaptor sequences and the low-quality raw reads were trimmed and discarded using Trimmomatic (v0.32) with default settings[1]. This procedure filtered out 3.56% of the raw reads on average and the coverage of reads per sample was on average 130. The trimmed reads were aligned on the most recent reference genome of E. coli K-12 MG1655 (NC_000913.3) by using bowtie2 (v2.1.0) that is designed to be fast and sensitive for reads longer than 50bp[2]. The average of alignment rate was 99.5%. Post-alignment quality control was performed using Picard to identify duplicated reads that were processed in the following step. Variant calling was performed using GATK toolkit[3], following its suggested pipeline called “best practices workflow”. To be brief, realignment (IndelRealigner), and variant calling (UnifiedGenotyper) were serially processed with its default settings. Finally, the quality of the called variants was assessed and low-quality variants were discarded using VariantFiltration and SelectVariants in GATK toolkit with default settings, which renders on average 5.2 variants (2.4 SNPs and 2.7 indels) per sample.

Supplementary tables

Supplementary Table 1. The groups that have 3 or more unique stresses in it.

strains	media	# of	#stresses	stresses
MG1655	M9	97	22	Chloramphenicol,Doxycycline, Trimethoprim, Spiramycin, Clindamycin, Kanamycin, Tetracycline, Tobramycin, Lomefloxacin, Nitrofurantoin, Piperacillin, foxitin, Nalidixic acid, Fusidic acid, Ampicillin, Spectinomycin, Streptomycin
K12	MS-Minimal	47	9	Ciprofloxacin, Doxycycline, Erythromycin, foxitin, Kanamycin, Nalidixic Acid, toin, Tetracycline, Tobramycin
MG1655	M9	5	5	, Osmotic, Butanol, H2O2, Acid
BW25113	MS-Minimal	11	3	Streptomycin, Tobramycin, Kanamycin

Supplementary Table 2. Genome sites mutated, number of replicates and generation elapsed under each stress.

Stress	Genome sites	No. of	Category	Generations
Nitrofurantoin	*rrrD, ylbE, mprA, nfsA, tsx-yajI, nfsB, cusA, marB-yobH, nmpC, essD, rpoA, ompR-rzpD, tfaD, rzoD, cusC, borD, ybcV, cusS, ylcI, nohD, aaaD, cusR, ybcY, tfaX, ompT, pauD, envY, ybcH, nfrA, nfrB, cusB, pheP, ybdG, ybdF, ybdJ, ybdK, rph, motB, insK-glyS*	4	1	302～353
Amikacin	*fusA, cydA, sbmA, holE, yaiT, trkH, yaiV, yaiW, atpA, rpoC, pyrE-rph, ylbE*	4	2	232～280
Chloramphenicol	*gyrB, marR, isrC, ompR, mdfA, rob, rplD, lon, ybiG-mdfA, soxR, atpB, atpI, yabT, fusA, sbmA, yaiT, trkH, yaiV, cpxA, ampH,*	9	2	211～441
Clindamycin	*pyrE-rph, prmB, rpmG, rph, rplB, ygbI-rplV*	4	2	391～396
Doxycycline	*acrR, hiuH, marR, fis, lpxM, rpoB, manY, lon, acrR, yhdP, marR, acrS, rph, paaA*	9	2	211～378
Erythromycin	*fis, acrB, yhdJ, ylbE*	2	2	340～340
Fusidic acid	*ylbE, fusA, ynfE*	4	2	277～358
Kanamycin	*rph, ampD, fusA, cyoA, fis*	2	2	403～403
Spectinomycin	*pyrE-rph, rph, rplB, yqgP, rpsE, fdrA-ylbF*	4	2	315～388
Spiramycin	*rlmN, rph, rplD*	4	2	348～370
Streptomycin	*hemB, rimP, rpsL, lysW, trkH, rsmG, pyrE-rph, cyoA, citG*	4	2	252～441
Tetracycline	*rsxD, mlaA, fucI, rph*	4	2	307～433
Tobramycin	*pssA, fusA, sbmA, yaiT, trkH, yiaO, yaiV, ampH, yaiW, yaiY, yaiZ, ddlA, iraP, phoA, yhiJ, fis, cyoB, pyrE-rph, rph, ylbE*	4	2	403～403
Sulfamethaxozole	*icd, folM, folP, folX, hemF*	4	3	315～328
Sulfamonomethoxine	*folM, ompC, ydgC, mprA, ynfL, pntB, mlc, pntA, clcB, folM, ynfK, ydaC, mdtJ, ynfM, ydaU, ydaD, mdtI, tasA, ydaH, rstA, rstB, ompR*	4	3	302～403
Ampicillin	*acrB, ftsI, envZ*	4	4	315～320
Piperacillin	*ftsI, envZ, frdD, rph, ompR*	4	4	297～454
Ciprofloxacin	*acrR, ompF, gyrA, ompF, sseA, rph, gyrB*	4	5	292～423
Lomefloxacin	*acrR, gyrA, marR, leuW*	4	5	292～353
Nalidixic acid	*gyrA, ychO*	4	5	302～312
Trimethoprim	*acrA, gyrB, rpoB, folA, kefC-folA, folA, pepT, roxA*	9	5	153～292

Supplementary Table 3. The performance of the ensemble predictor with the rare class oversampled. The rare class was oversampled, reaching various percentage of the whole dataset.

Ratio (%)	No oversampling	20	30	40	50
AUC	0.963	0.942±0.034	0.939±0.039	0.938±0.041	0.939±0.038
AUPRC	0.374	0.391±0.042	0.388±0.049	0.393±0.045	0.389±0.047

Supplementary Table 4. *P-value*s for the statistical significance of the difference in the performance of the ensemble predictor and each individual predictor.

P value	ANN	NB	SVM
Ensemble	5×10^{-14}	4×10^{-16}	0
ANN		0.26	0
NB			0

Supplementary Table 5: The combinations that were evaluated for the hyper-parameters of the ANN: number of layers, number of nodes in each layer and dropout rate. The last column, Count, describes the number of genome sites for which such a setting is optimal.

**Index**	**Number of layers**	**Number of nodes in each layer**	**Dropout rate**	**Count**
1	2	57_37	0.4	257
2	1	54	0.23	213
3	1	78	0.14	144
4	1	26	0.28	110
5	2	54_12	0.33	98
6	1	4	0.42	95
7	1	63	0.44	88
8	2	49_40	0.22	84
9	1	33_	0.42	80

10	1	82	0.32	79
11	2	11_32	0.27	72
12	2	43_4	0.06	70
13	2	17_11	0.27	66
14	2	72_35	0.35	61
15	1	41	0.34	54
16	1	32	0.27	53
17	2	45_12	0.38	49
18	2	75_27	0.06	45
19	1	2	0.33	36
20	1	77	0.25	34
21	2	17_7	0.25	34
22	2	68_22	0.09	28
23	1	17	0.3	24
24	1	39	0.07	22
25	2	4_15	0.26	17
26	1	31	0.01	16
27	2	56_5	0.34	13
28	2	19_21	0.26	11
29	2	2_27	0.3	10
30	2	56_3	0.36	10
31	1	26	0.47	9
32	2	35_34	0.23	8

Supplementary Table 6. The performance of ANN for predicting a sample set of the genome-sites (100) using different optimization method and activation function for the hidden units.

	**Optimization method**	**Mean**	**Std**
AUC	Adagrad	0.898829	0.116439
	adam	0.907134	0.110569
	RMSProp	0.882032	0.107417
AUPRC	Adagrad	0.309942	0.204843
	adam	0.311344	0.198568
	RMSProp	0.290311	0.191896
	**Activation function**	**Mean**	**Std**
AUC	relu	0.880314	0.123222
	sigmoid	0.878872	0.095093
	tanh	0.910485	0.109351
AUPRC	relu	0.282977	0.218579
	sigmoid	0.278235	0.190614
	tanh	0.290601	0.197809

Supplementary Table 7. The performance of ANN for prediction the 1,990 genome sites with one hyperparameter of the optimal setting changed (The predictions were merged and one AUC and one AUPRC were generated).

	Optimal	Relu	Sigmoid	Adagrad	RMSProp
AUC	0.946	0.932	0.927	0.942	0.938
AUPRC	0.324	0.308	0.297	0.317	0.313

Supplementary Table 8. Facts about the sequencing data for validating the prediction performance.

**Sample ID**	**# of forward reads in raw file1**	**# of reads after preprocessing**	**Coverage after preprocessing**	**Alignment rate (%)**	**# of called variants (SNPs/Indels)**
1	4650618	4522114	194	99.69%	6(2/4)

2	1772924	1721907	74	99.48%	7(4/3)
3	2489673	2422217	104	99.61%	7(3/4)
4	2529148	2459245	106	99.59%	4(2/2)
5	3043442	2947585	127	99.63%	6(3/3)
6	2400443	2328544	100	99.64%	3(1/2)
7	1850801	1803351	77	99.54%	5(2/3)
8	2143281	2085257	89	99.50%	3(1/2)
9	1614838	1574826	67	99.58%	4(2/2)
10	3376379	3272405	141	99.53%	4(2/2)
11	2102595	2043992	88	99.63%	4(2/2)
12	2065844	1696206	73	97.05%	4(3/1)
13	9650868	9272859	399	99.61%	4(1/3)
14	4493859	4310317	185	99.66%	3(2/1)
15	7114137	6837946	294	99.64%	6(1/5)
16	981488	955251	41	99.62%	7(4/3)
17	2245489	2150450	92	99.06%	4(3/1)
18	1795273	1730752	74	99.56%	4(3/1)
19	2379489	2322111	100	99.57%	5(1/4)
20	2508383	2424148	104	99.65%	8(3/5)
21	1742637	1696797	73	99.41%	7(4/3)
22	2197585	2136841	92	99.66%	5(3/2)
23	4232282	4102622	176	99.60%	4(3/1)
24	2142538	2026437	87	99.43%	6(1/5)
25	1658535	1619400	69	99.59%	6(2/4)
26	1346701	1299520	56	99.53%	5(3/2)
27	3065908	2973177	128	99.65%	5(1/4)
28	3741911	3645478	157	99.65%	4(3/1)
29	2166803	2101503	90	99.54%	8(4/4)
30	2855256	2767378	119	99.70%	8(5/3)
31	6045106	5860875	252	99.65%	4(3/1)
32	3226050	3143812	135	99.33%	5(1/4)

33	3300525	3194287	137	99.65%	3(2/1)
34	4319966	4161577	179	99.64%	8(2/6)
35	6752890	6504107	280	99.68%	5(3/2)

Supplementary figures

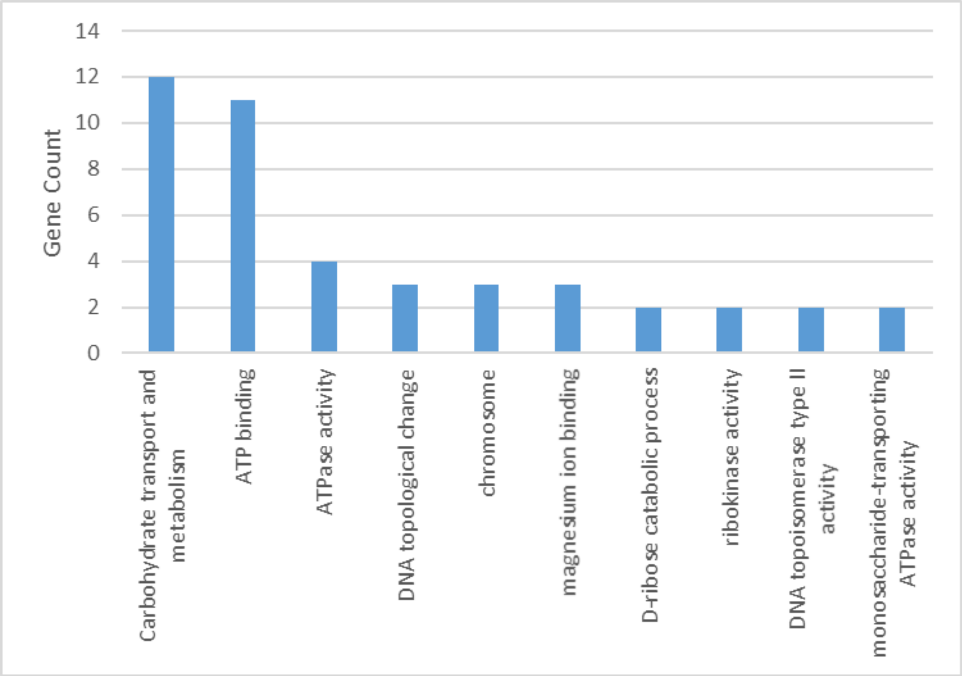

Supplementary Fig. 1. David analysis of the top mutated genes found in the database.

Summary of the top functions and pathways of the top 20 genes most hit by mutations. Carbohydrate transport was found in 12/20 genes.

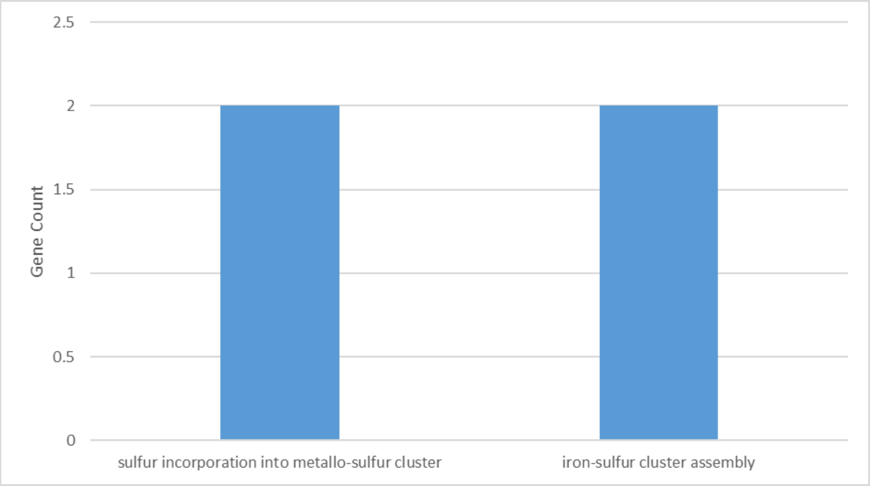

Supplementary Fig. 2. David analysis of the genes found in the hotspots. Only two pathways were enriched related to sulfur processes.

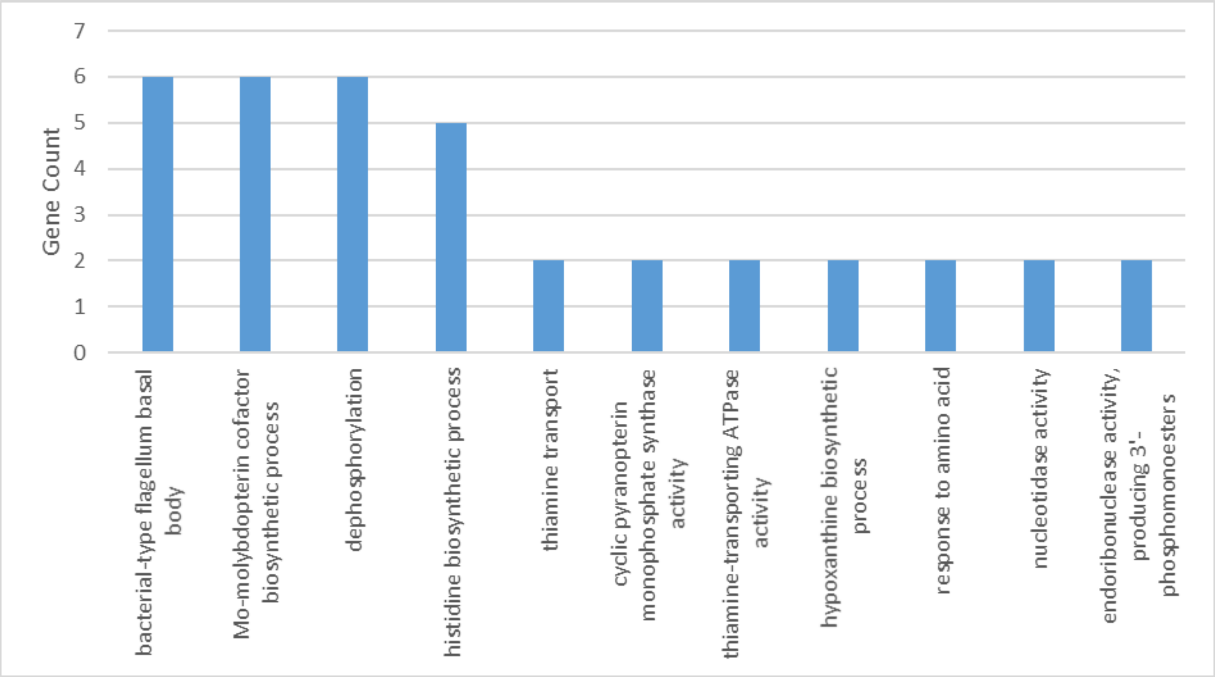

Supplementary Fig. 3. David Analysis of the genes found in the coldspots. Most of the pathways hits are related to cell structures (flagella) and aminoacids (synthesis and response).

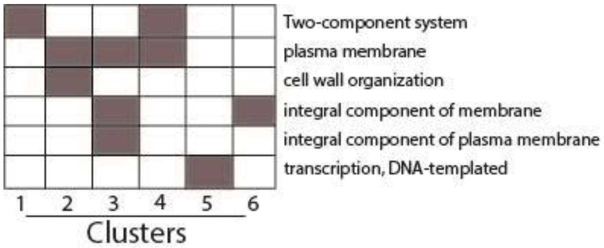

Supplementary Fig. 4. Summary of the function enrichment of the clusters. The only pathway enriched is Two Component Systems. The pathways and GO terms with a P-value < 0.1 and found in at least 10% of the genes present in each cluster.

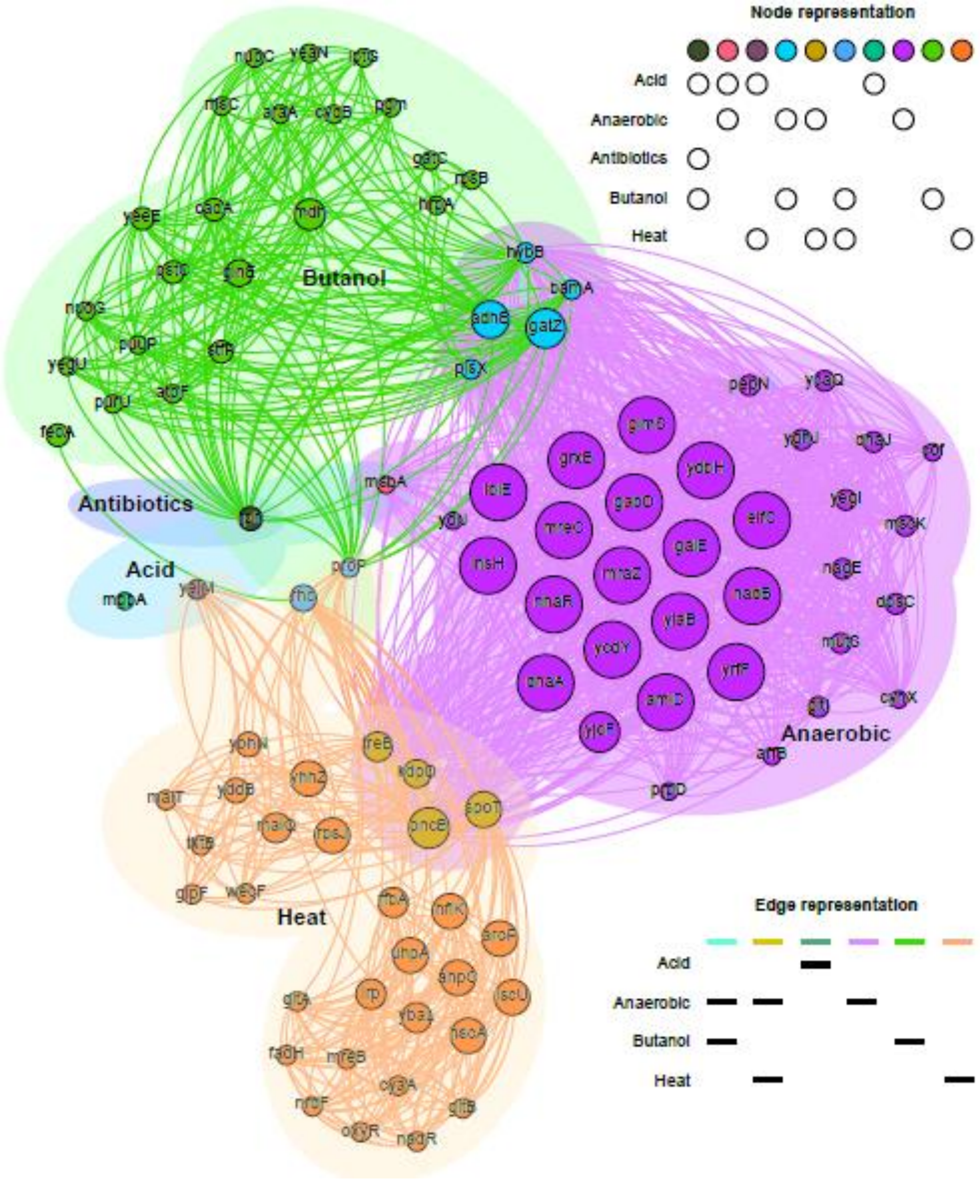

Supplementary Fig. 5. Network of genes adapted under five most popular stresses. Node represents gene mutated under one or more stresses and edge between two nodes represents co−occurrence of two nodes under one or more stresses. Node size is proportional to the mutation ratio of a gene. And thickness of edge is proportional to the occurrence ratio of two adjacent nodes. If a gene is mutated under more than one stress, then we take an average of mutation ratios for all stresses involved. If an edge is occurred in more than one stresses, then we take an average of mutation co-occurrence ratios for all stresses involved. Node color represents a list of stresses under which the mutated gene was adapted and edge color represents a list of stresses under which the two mutated genes were together

.

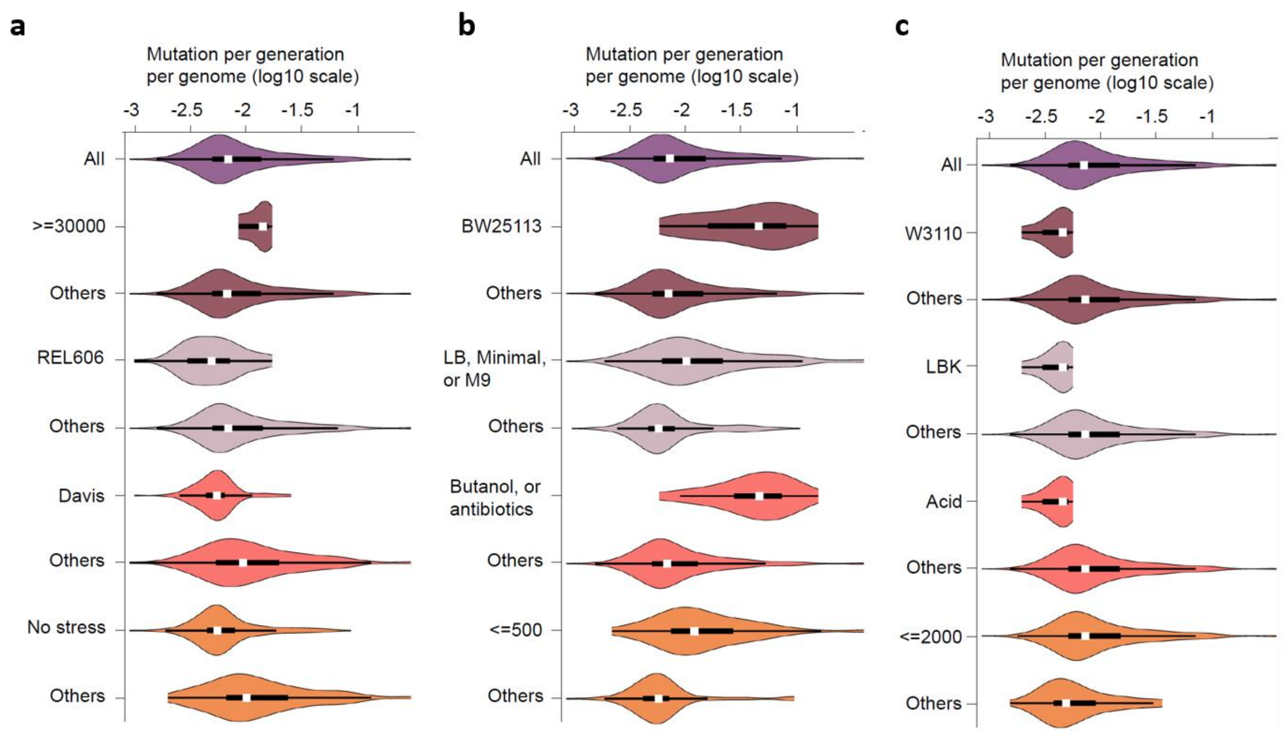

Supplementary Fig. 6. The distribution of mutation rate. Color encodes a way of splitting all the evolution runs into two groups according to one of the factors, generation, strain, medium and stress, regardless of other factors. Only violin plots in the same color within each panel are comparable. Each violin plot displays the distribution of mutation rate for all the evolution runs (All) or a subgroup. The white dot, the thick bar and the thin bar in each plot represents median and interquartile range and 95% confidence interval. The panels **a, b** and **c** are for investigating the effect of generations, strain BW25113, and strain W3110 on mutation rate respectively.

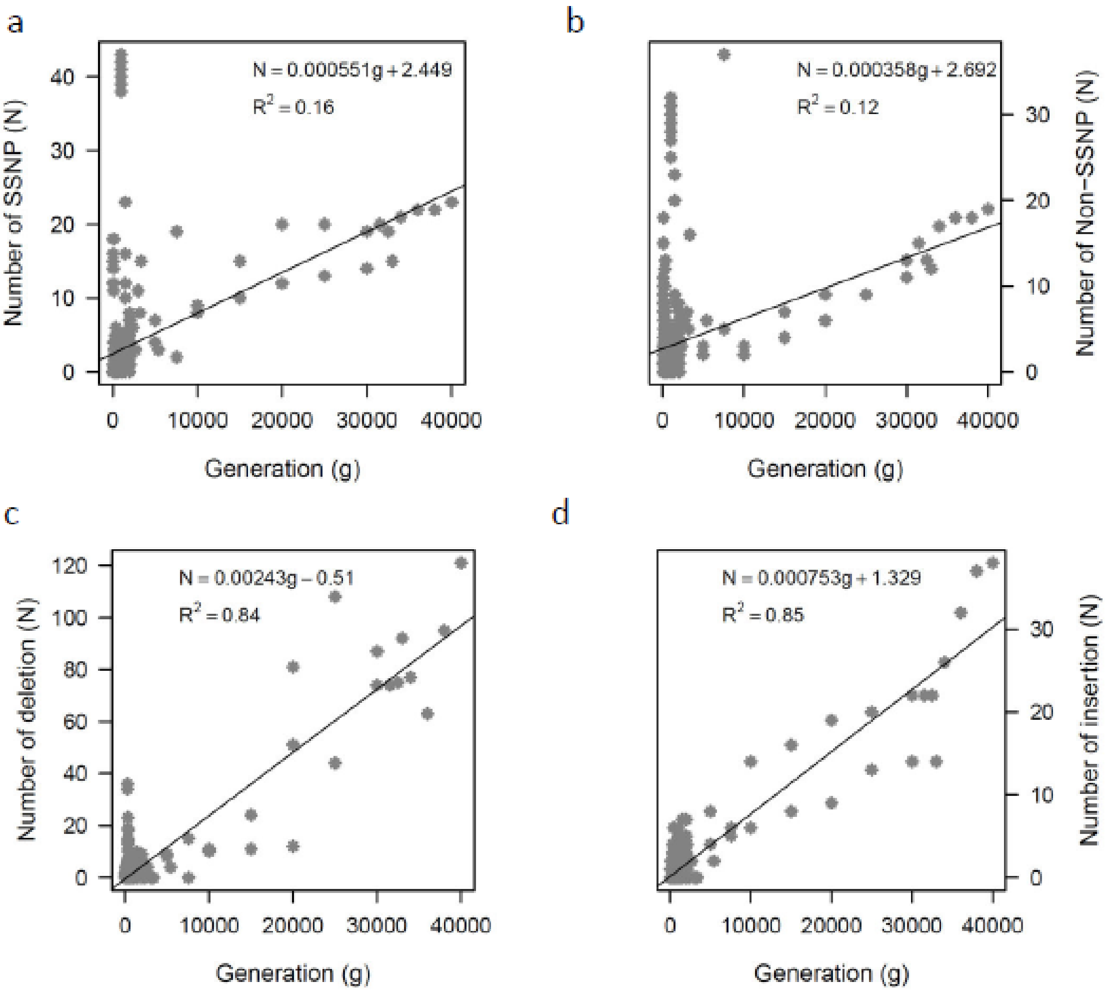

Supplementary Fig. 7. The number of synonymous mutations (**a**) non-synonymous mutations (**b**) deletion (**c**) and insertion (**b**) as a function of generations elapsed. All the mutator strains were excluded when conducting such analysis.

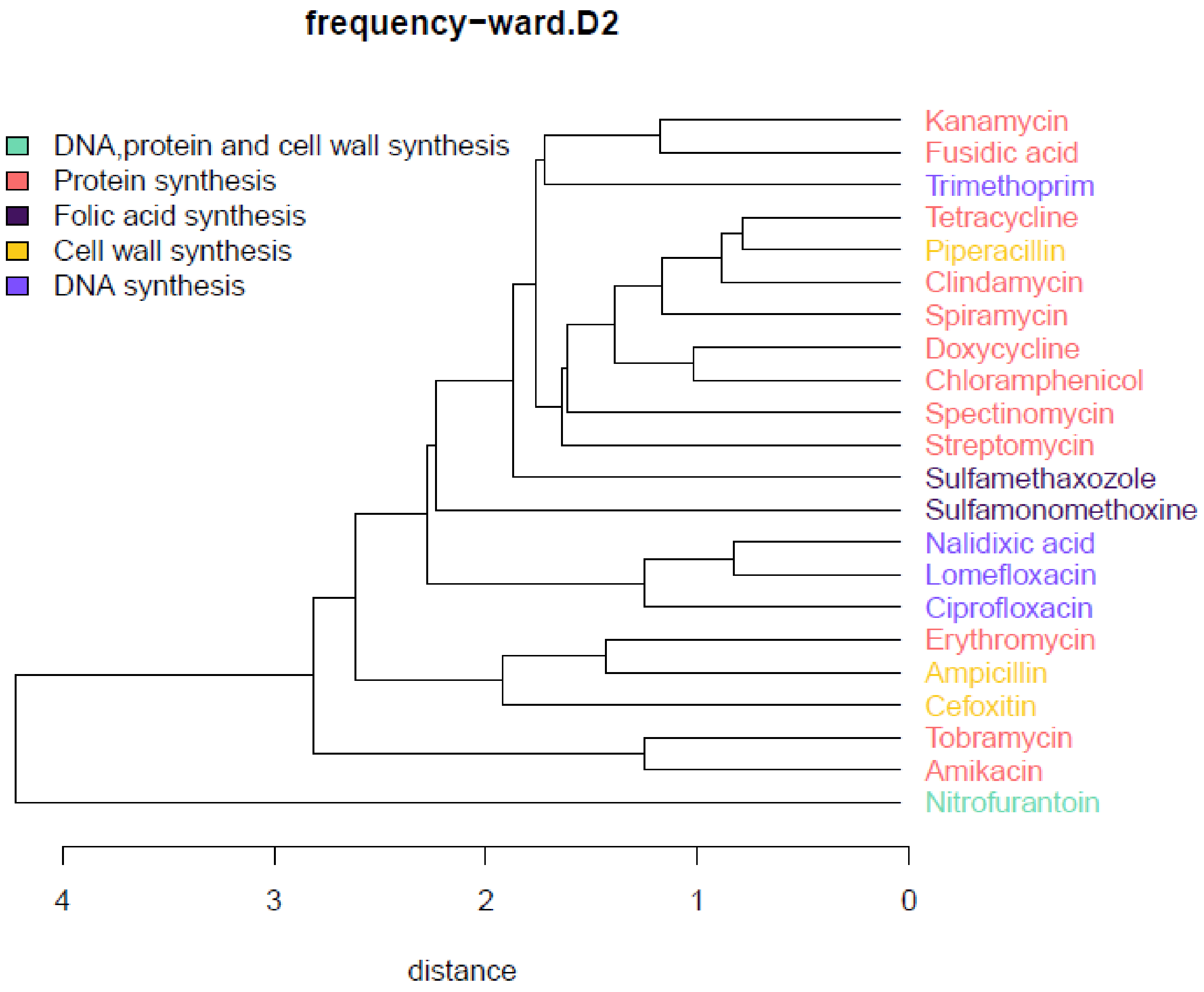

Supplementary Fig. 8. A dendrogram illustrating the clusters of antibiotics generated by hierarchical clustering. The legend describes the action mechanism of each category of antibiotics. The antibiotics in each category shared the same color in the dendrogram (Kim, 2015). When computing the pairwise distance between mutation profiles under various antibiotics, mutation frequency profiles were used.

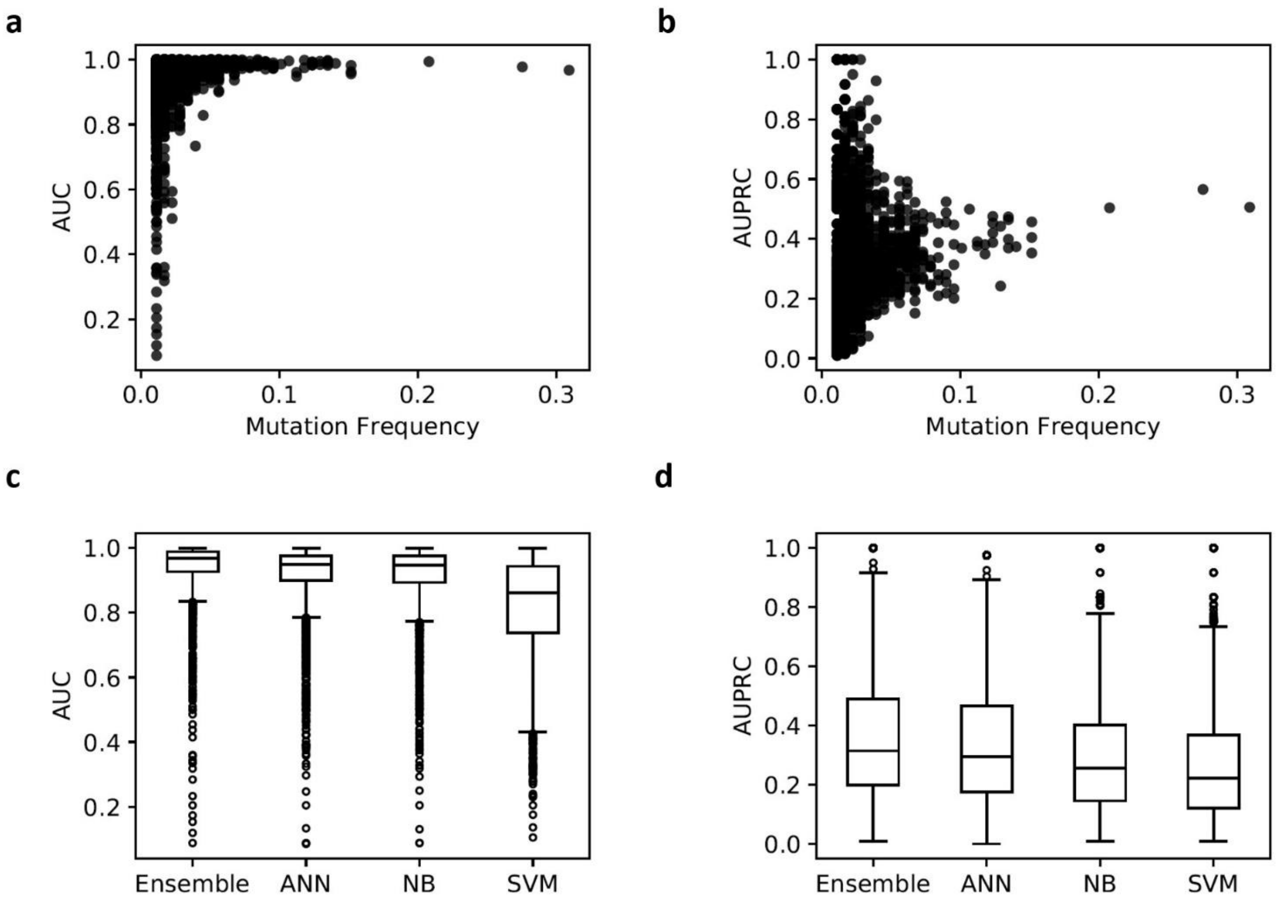

Supplementary Fig. 9 Prediction performance and mutation frequency (a, b) The performance of the ensemble predictor when predicting mutations on the 1990 genome sites versus the frequency of a genome site being mutated under different culture conditions. (**c, d**) The distribution of the performance of the ensemble predictor and each individual predictor. The upper whisker and lower whisker are equal to the upper quantile+1.5 IQR and lower quantile-1.5IQR, where IQR is equal to upper quantile – lower quantile.

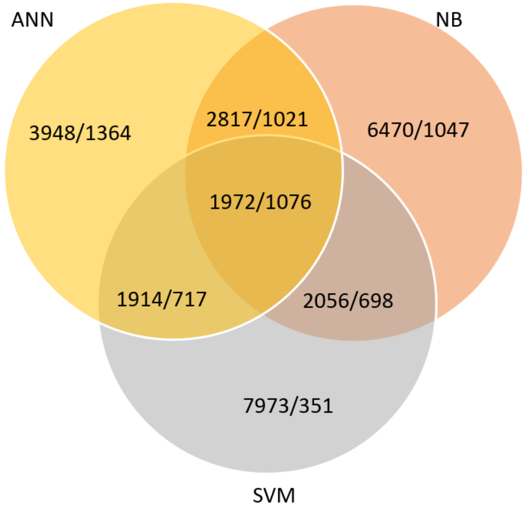

Supplementary Fig. 10. A Venn diagram showing the intersection among the predicted mutations by three predictors: ANN, NB, SVM. In the notation a/b, a represents the number of predicted mutations and b denotes the number of True Positives.

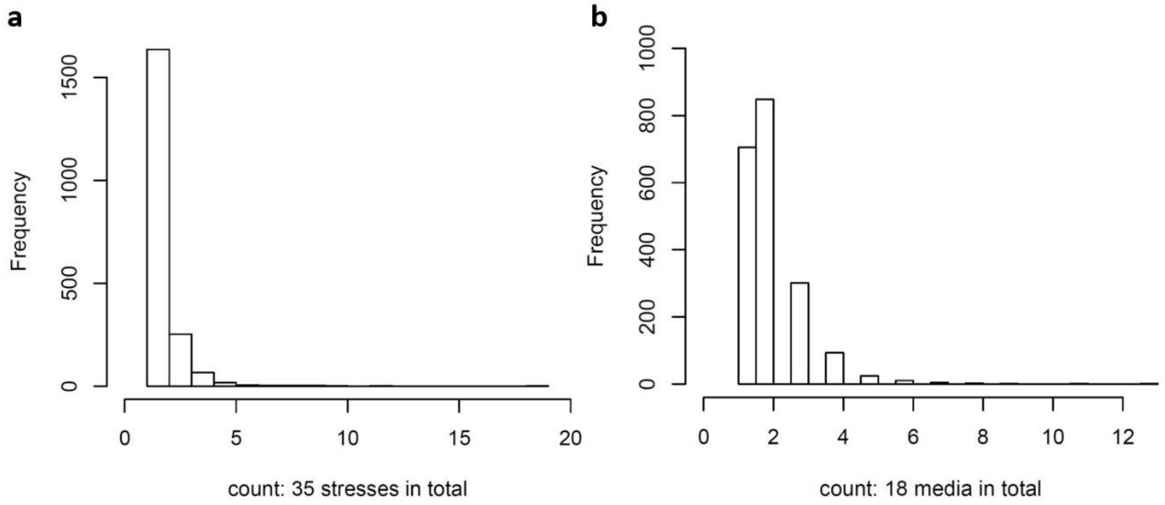

Supplementary Fig. 11. The generality and specificity of mutations with respect to stresses and media. (**a**) The distribution of the number of unique stresses per mutation target. (**b**) The distribution of unique media per mutation target.

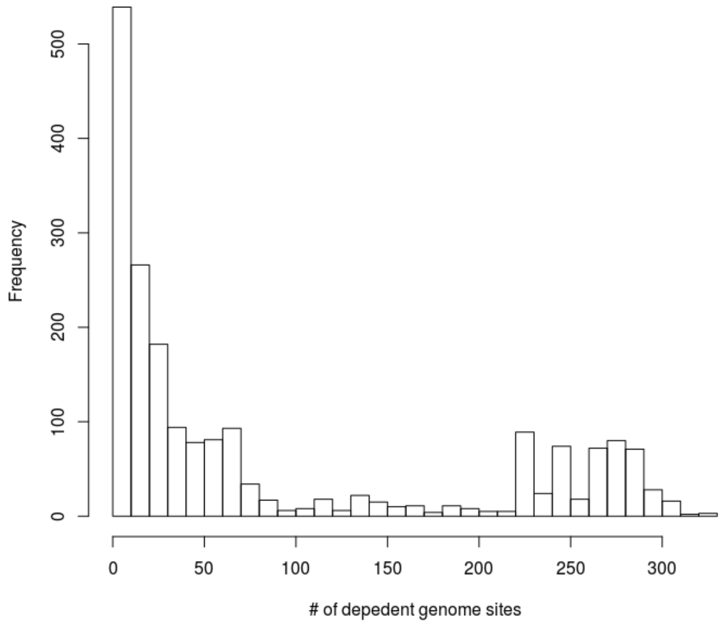

Supplementary Fig. 12. A histogram of the number of dependent genome sites for each genome site (1990 genome sites in total). For each genome site, the number of genome sites significantly dependent on that gene was computed by chi-square test. The cutoff for the P-value is 0.05.

Supplementary references

1. Bolger, A. M., Lohse, M. & Usadel, B. Trimmomatic: a flexible trimmer for illumina sequence data. Bioinforma. 30, 2114–2120 (2014).

2.Langmead, B. & Salzberg, S. L. Fast gapped-read alignment with bowtie 2. Nat. methods 9, 357 (2012).

3.McKenna, A. et al. The genome analysis toolkit: a mapreduce framework for analyzing next-generation dna sequencing data. Genome research 20, 1297–1303 (2010).
