# Supplementary material for: Predicting the evolution of Escherichia coli by a data-driven approach
Source: Nat Commun. 2018 Sep 3;9:3562. doi: 10.1038/s41467-018-05807-z (PMC6120903; doi:10.1038/s41467-018-05807-z)
Supplement: Supplementary file 3 — Description of Additional Supplementary Files [file 41467_2018_5807_MOESM3_ESM.pdf]

Description of Additional Supplementary Files

File Name: Supplementary Data 1
Description: A list of publications used for constructing the database (Supplementary Data 1.xlsx).

File Name: Supplementary Data 2
Description: MutationDB v1.0 main Database file (Supplementary Data 2.xlsx).

File Name: Supplementary Data 3
Description: The mutation frequency of each genome site across the 178 unique experimental settings (Supplementary Data 3.xlsx).

File Name: Supplementary Data 4
Description: The genes, corresponding GO terms and pathway information for each of the 6 clusters identified during the GO analysis database (Supplementary Data 4.xlsx).

File Name: Supplementary Data 5
Description: Co-occurrence mutation analysis (Supplementary Data 5.xlsx).

File Name: Supplementary Data 6
Description: A list of on genes governing DNA repair, DNA supercoiling or DNA assembling or encoding DNA polymerase, DNA gyrase, DNA glycosylase and DNA repair proteins (Supplementary Data 6.xlsx).

File Name: Supplementary Data 7
Description: The mutation rate calculated for all strains in the database (Supplementary Data 7.xlsx).

File Name: Supplementary Data 8
Description: Feature frequencies for individual predictor on all the 1,990 genome sites (Supplementary Data 8.xlsx).

File Name: Supplementary Data 9
Description: The predictions by ANN, SVM, NB and the Ensemble predictor in leave-onecondition-out cross validation (Supplementary Data 9.xlsx).

File Name: Supplementary Data 10
Description: The mutation prediction results of a novel condition (osmotic NaCl stress, MG1655 strain under M9 salt media) by the three predictors using bootstrapping (Supplementary Data 10.xlsx).

File Name: Supplementary Data 11
Description: Ranked list of mutation targets with respect to coverage across different stresses and media (Supplementary Data 11.xlsx).
